# Pre-hospital cooling in community-acquired heat stroke (CAHS): evidence, challenges, and strategies

**DOI:** 10.1186/s40001-025-02628-x

**Published:** 2025-06-11

**Authors:** Shu Cong, Guangxin Zheng, Xiaojuan Liang, Jianjun Gui, Hong Zhang, Jun Wang

**Affiliations:** 1 Department of Emergency Medicine, Bao’an Clinical Institute of Shantou University Medical College, Shenzhen, China; 2 Shenzhen Center for Prehospital Care, Shenzhen, China; 3 Department of Critical Care Medicine, Bao’an Clinical Institute of Shantou University Medical College, Shenzhen, China

**Keywords:** Heat stroke, Prehospital emergency care, Hyperthermia, Cooling methods

## Abstract

**Background:**

Heat stroke (HS) is a critical medical emergency characterized by severe hyperthermia and central nervous system dysfunction, occurring predominantly in conditions of high ambient temperatures or through physical exertion. With rising global temperatures, the incidence and severity of HS are expected to increase, presenting substantial public health challenges.

**Objectives:**

This review synthesizes current evidence on cooling methods for HS, addresses challenges in managing community-acquired heat stroke (CAHS), and proposes strategies to enhance pre-hospital and emergency department (ED) care.

**Methods:**

We evaluated existing literature on cooling strategies, focusing on different subtypes of HS (classic and exertional) and the efficacy of rapid cooling techniques. In addition, we reviewed epidemiological data and proposed a subclassification of HS into supervised and community-acquired heat stroke to better understand healthcare accessibility impacts.

**Results:**

Water, particularly cold water immersion, is recognized as the most effective medium for cooling HS due to its superior thermal properties. Rapid cooling (> 0.15 °C/min) is crucial for survival, significantly reducing case fatality rates and medical complications. Supervised HS, often managed promptly at athletic or military events, shows near-zero fatality rates with immediate cooling. In contrast, CAHS presents unique challenges due to delayed medical intervention and lack of immediate cooling resources. Current strategies to improve CAHS management include public education, dispatcher-guided first aid, and pre-hospital cooling techniques such as internal cooling with cold saline and gastric lavage.

**Conclusions:**

Effective HS management hinges on rapid cooling, with water immersion being the gold standard. To mitigate the rising burden of HS, particularly in community settings, there is a need for enhanced public awareness, training for emergency responders, and improved pre-hospital equipment. Future strategies should focus on integrating cooling interventions into emergency response protocols and ensuring timely access to cooling resources in both pre-hospital and ED settings.

## Introduction

Heat stroke (HS) is a life-threatening medical emergency characterized by a critical imbalance between thermogenesis and heat dissipation [1]. It is most commonly triggered by strenuous physical activity or prolonged exposure to high ambient temperatures, leading to a core body temperature exceeding 40.5 °C. HS carries substantial morbidity and mortality risks, yet robust epidemiological data remain limited. Most existing research focuses on specific subpopulations, such as athletes and military personnel [2–4], with few studies addressing its burden in the general population. For example, United States emergency department (ED) data from 2009 to 2010 estimated approximately 8251 HS-related visits annually, with a hospitalization rate of 54.6% and an in-hospital mortality of 3.5% [5]. However, these figures likely underestimate the true incidence, excluding pre-hospital deaths and misdiagnosed cases.

The global climate crisis is expected to magnify the public health impact of HS. Heat-related mortality in individuals aged ≥ 65 years has increased by approximately 68% over the past two decades, with more than one-third of heat-related deaths in the last 30 years attributed to climate change [6]. In 2018 alone, an estimated 296,000 deaths worldwide were linked to extreme heat exposure [7]. Similarly, the summer of 2022 saw approximately 61,672 heat-related deaths across Europe [8]. In 2023, a notable rise in heat-related illnesses (HRIs) was reported globally, including 119,605 HRI-related ED visits documented across selected regions of the United States [6, 9].

Diagnosis of HS is primarily clinical and hinges on three cardinal features: hyperthermia, central nervous system (CNS) dysfunction (e.g., ataxia, seizures, altered mental status, or coma), and a history of recent exposure to either high environmental temperatures (classic heat stroke, CHS) or intense physical exertion (exertional heat stroke, EHS). Additional common signs include tachycardia, tachypnea, and hypotension [1].

HS is broadly classified into two major subtypes [1]: CHS and EHS. While both share a common pathophysiologic mechanism of impaired thermoregulation and resultant heat accumulation, their etiologies differ. CHS typically occurs from passive heat exposure, often in vulnerable populations with diminished thermoregulatory capacity. In contrast, EHS arises from excessive endogenous heat production during vigorous physical activity that overwhelms the body’s cooling mechanisms.

HS represents a true time-sensitive emergency. The therapeutic window is narrow—estimated at approximately 30 min—within which prompt recognition and rapid initiation of cooling are imperative to prevent irreversible organ damage [10]. The duration of hyperthermia above the cellular injury threshold is a critical determinant of clinical outcomes. Accordingly, the primary goal of treatment is to reduce core body temperature to below 40 °C within this brief therapeutic window.

Timely initiation of cooling is strongly associated with improved outcomes [3, 4, 11, 12]. To better reflect the influence of healthcare accessibility and response time, we propose a clinically meaningful sub-classification of HS into supervised heat stroke (SHS) and community-acquired heat stroke (CAHS). SHS refers to cases occurring in settings, where medical personnel are present and can promptly initiate treatment, such as athletic events or military training. Conversely, CAHS occurs in community settings, where immediate medical assistance is not readily available, primarily affecting the general population.

This review synthesizes evidence on cooling methods, examines challenges in CAHS management, and proposes strategies to enhance pre-hospital and ED care, emphasizing real-world applicability in the context of climate-driven HS surges.

## Evidence on cooling in heat stroke

### Water as the optimal cooling medium

Water is widely recognized as the most effective medium for cooling in HS. Casa et al. [10] emphasized the superior thermal properties of water in facilitating heat dissipation, establishing it as the gold standard for HS treatment. Key properties include:

1. High thermal conductivity: water (630.5 mW/m^2^ K) is approximately 24 times more efficient in heat transfer than air (26.2 mW/m^2^ K).
2. High specific heat capacity: water absorbs substantial amounts of heat with minimal increases in temperature.
3. High density: at 0.9922 g/cm^3^, water is significantly denser than air (0.0012 g/cm^3^), enhancing its heat absorption capacity.
4. Rapid cooling rate: water can cool the body approximately four times faster than air at the same temperature.
5. Extensive contact surface: submersion ensures nearly 100% body surface area contact, maximizing conductive heat loss.
6. Favorable physiological response: cold water immersion reduces peripheral vasoconstriction and shivering in hyperthermic individuals, further promoting heat loss.

Based on these characteristics, cold water immersion is widely endorsed as the first-line treatment for HS.

### Cooling rates and outcomes in EHS

Erica et al. [13] conducted a systematic review of 32 studies involving 521 EHS patients to investigate the relationship between cooling rate and case fatality. The analysis revealed that achieving a cooling rate > 0.15 °C/min was critical for survival. Patients cooled above this threshold had a 0% case fatality rate, whereas those cooled at slower rates exhibited a 4.41% fatality rate.

Moreover, the incidence of medical complications was significantly higher in patients with insufficient cooling. Among survivors, the complication rate was 22.46% in the low-cooling group, compared to only 0.77% in the adequate-cooling group. These findings underscore the importance of rapid cooling and support the use of effective methods, such as continuous cold water dousing or full-body immersion. In contrast, ineffective techniques—including ice packs to arterial sites, spray fans, cold IV infusions, cooling blankets, warm water wiping, or single-fan use—were associated with lower cooling rates and worse outcomes.

### Supervised EHS: case fatality reduction to near zero

Demartini et al. [3] reported outcomes from 274 EHS cases among approximately 130,000 long-distance runners over 18 years, demonstrating a 0% case fatality rate when cold water immersion (10 °C) was initiated within 2 min. Similarly, Benjamin et al. [4] observed a case fatality rate of < 2% among 48 EHS cases in a military cohort of 2.6 million personnel, with 70% receiving pre-hospital cooling.

These findings reinforce the critical role of early cold water immersion in EHS management. This approach has been adopted by the International Olympic Committee’s Expert Working Group on Adverse Weather Impacts [14, 15] and incorporated into pre-hospital treatment protocols for the Tokyo 2020 Olympics and Paralympics, as well as into expert guidelines for EHS prevention in American high schools [16].

### Supervised CHS: case fatality reduction to 5.6%

Yezli et al. [17] conducted a systematic review of 44 studies including 2,632 patients with CHS, predominantly in medically supervised contexts associated with religious gatherings. The mean patient age was 57.4 years (range: 4 days to 97 years), with a high prevalence of comorbidities, such as obesity, diabetes, and cardiovascular disease.

Most patients received pre-hospital cooling—primarily via evaporative methods—with limited use of cold water immersion. The overall case fatality rate was 5.6%, substantially lower than the 58% reported during the 2003 heatwave in Lyon, France [11], and the 26.5% among community-acquired EHS cases in China [12].

## Challenges in pre-hospital cooling for CAHS patients

CAHS presents distinct challenges not typically encountered in SHS. In SHS, medical personnel or trained volunteers are often present to initiate immediate cooling. In contrast, CAHS typically arises in isolated settings or among bystanders lacking the knowledge or resources to administer first aid. Consequently, CAHS patients are reliant on emergency medical services (EMS), which may take 10–30 min to arrive in urban areas and even longer in rural regions.

Ambulances often lack adequate cooling equipment due to space and logistical constraints [18]. Even upon ED arrival, delays in diagnosis and treatment may occur due to limited familiarity with HS management among emergency staff, leading to further delays in initiating effective cooling [19, 20].

## Strategies to improve CAHS management

*Bystander cooling*: With the number of days featuring dangerously elevated temperatures projected to increase in the coming years [6–9, 21], public education on HS recognition and first aid should be integrated into existing emergency response training (e.g., CPR, trauma care, hemorrhage control) [22]. This requires sustained policy and financial support, with long-term benefits in outcomes.

*Dispatcher-guided cooling*: Emergency dispatchers should be trained to recognize HS risk based on patient location and activity. They can instruct bystanders to assess mental status and temperature and to initiate basic cooling [23]—such as relocating the patient to a shaded area, applying fans, or pouring chilled water over the body using locally available resources (e.g., 10–20 bottles of 500 mL cold beverages).

### Cooling in the ambulance

In regions and seasons with a high incidence of heat stroke (HS), ambulances should be appropriately equipped with cooling materials, such as ice water, chilled saline, or ice packs [23]. Given the spatial limitations of ambulances, immersion in a cold-water bath—the gold standard for SHS treatment—is impractical. Therefore, optimizing the use of cold water through alternative modalities, particularly internal cooling, is essential.

A practical and effective approach comprises the use a semiconductor vehicle-mounted refrigerator, which is small in size, convenient to carry, and can maintain a temperature control between 5 and 10 ℃. Inside the refrigerator, there are 20 bottles of 500-mL normal saline for infusion stored. Of the total 10,000 mL available, 8000 mL can be used for repeated whole-body wiping, and the remaining 2000 mL can be rapidly administered intravenously via three peripheral venous lines. Rapid infusion of cold saline at 30 mL/kg, combined with 20 mg of intravenous diazepam, has been shown to reduce core body temperature by 1.5 ± 0.2 °C in conscious, normothermic volunteers without inducing adverse cardiovascular or respiratory effects [24].

Further enhancement of cooling efficiency may be achieved through gastric lavage using ice-saline. This method involves gastric infusion of 500 mL of ice-saline water via a gastric tube using a 60 mL syringe, allowing a dwell time of 5 min before withdrawal, and repetition with another 500 mL. This technique has demonstrated cooling rates up to 2.6 °C/hour and is particularly valuable in settings, where external cooling is constrained [25].

In pre-hospital settings, rectal temperature monitoring—the gold standard—may not be feasible due to privacy concerns and logistical limitations. In such cases, tympanic thermometry offers a hygienic, rapid, and practical alternative. There is no need to delay cooling while awaiting rectal temperature confirmation [23, 26].

### Cooling in the ED

When the critical 30-min window for pre-hospital cooling is missed, immediate and aggressive cooling in the ED becomes imperative to improve outcomes. By the time HS patients arrive at the ED, 30–60 min may have elapsed since collapse, underscoring the urgency of intervention.

Upon arrival, rapid assessment must be followed by prompt initiation of cooling. The ED environment is conducive to the preparation and administration of ice-water immersion, which remains the most effective method. Immersion in ice water at 2 °C can achieve a cooling rate of at least 0.2 °C per minute, enabling the rapid reduction of core temperature to below 40 °C.

Crucially, life-saving cooling should never be delayed for diagnostic procedures, such as tracheal intubation or computed tomography. Delays in cooling are associated with increased risk of multi-organ dysfunction and death. Rectal temperature monitoring is the gold standard. Where rectal temperature is unavailable, treatment protocols should be guided by immersion duration: patients may be immersed in ≤ 9 °C water for 11–12 min or in 10–26 °C water for 18–19 min, which has been shown to safely reduce core temperatures within therapeutic targets [27].

## Conclusion

Heat stroke is a time-critical emergency, where rapid cooling within 30 min determines survival. Cold water immersion remains the most effective method, achieving near-zero mortality in supervised settings. However, CAHS faces significant barriers due to delayed recognition and limited pre-hospital cooling access. To address this, public education, dispatcher-guided protocols, ambulance preparedness, and streamlined ED interventions are essential. As climate change drives HS incidence, global health systems must prioritize these strategies to reduce morbidity and mortality.

## Data Availability

No datasets were generated or analysed during the current study.

## Abbreviations

CAHS: Community-acquired heat stroke
CHS: Classic heat stroke
CNS: Central nervous system
CWI: Cold water immersion
ED: Emergency department
EHS: Exertional heat stroke
EMS: Emergency medical services
HS: Heat stroke
HRIs: Heat-related illnesses
SHS: Supervised heat stroke
